# Integrative neurobiological mechanisms of acupuncture in post-stroke cognitive impairment: from neurotransmission to brain network remodeling

**DOI:** 10.3389/fneur.2026.1744242

**Published:** 2026-02-25

**Authors:** Wei Li, Lebin Liu, Weiwei Liu, Huiping Liu

**Affiliations:** Department of Rehabilitation Medicine, Hubei Rongjun Hospital, Wuhan, China

**Keywords:** acupuncture, BDNF signaling, brain network remodeling, neuroinflammation, neuroplasticity, neurotransmission, post-stroke cognitive impairment, systems neurobiology

## Abstract

Post-stroke cognitive impairment (PSCI) is a prevalent sequela of stroke that severely limits recovery and quality of life. Accumulating evidence indicates that acupuncture exerts significant neuroprotective and cognitive-enhancing effects in PSCI; however, the underlying mechanisms remain fragmented across molecular, cellular, and systems levels. This review proposes an integrative neurobiological framework linking neurotransmission, neuroinflammation, neurotrophic signaling, and brain network remodeling to explain how acupuncture promotes neurorepair and cognitive restoration after stroke. We systematically summarized recent clinical and experimental findings from 2001 to 2025 and categorized the converging mechanisms into five inter-related dimensions: (1) regulation of neurotransmitters and synaptic plasticity; (2) anti-inflammatory and immune modulation; (3) anti-oxidative stress and anti-apoptotic actions; (4) up-regulation of BDNF-related pathways and neurotrophic signaling; and (5) enhancement of neurogenesis and reconstruction of brain functional networks. Collectively, these multimodal effects form a systems-level cascade through which acupuncture may facilitate neuroplastic remodeling and cognitive recovery. Current challenges include heterogeneity of study design, insufficient multi-omics validation, and limited longitudinal imaging evidence. Future research should integrate molecular biomarkers, neuroimaging, and clinical outcomes to verify this multi-layered mechanistic framework and to guide precision acupuncture protocols for PSCI rehabilitation.

## 1 Introduction

PSCI is a common and disabling consequence of stroke, affecting nearly one-third of survivors and markedly compromising functional independence and quality of life (1). Despite advances in acute stroke management and secondary prevention, effective therapeutic strategies for PSCI remain limited (2). Pharmacological interventions such as cholinesterase inhibitors and memantine provide only modest benefit, underscoring the urgent need for complementary neurorehabilitative approaches that target multi-dimensional mechanisms of brain repair (3).

Acupuncture, a key modality of traditional Chinese medicine (TCM), has been increasingly recognized as a promising neurorehabilitative intervention for post-stroke deficits (4, 5). Accumulating clinical and experimental evidence demonstrates that acupuncture can enhance cognitive function, promote neuroplasticity, and modulate cerebral perfusion (6, 7). From the TCM perspective, PSCI corresponds to syndromes such as deficiency of kidney essence, disharmony of heart and spleen, or phlegm-stasis obstructing the orifices, all reflecting disrupted communication between the brain (sea of marrow) and viscera (8). In TCM, these classical syndromes emphasize internal imbalance and systemic dysfunction rather than localized pathology (9). Modern neurobiology provides parallel interpretations of these concepts: “deficiency of essence” resembles neurotrophic insufficiency and synaptic loss; “heart–spleen disharmony” parallels metabolic and neuroimmune dysregulation; and “phlegm-stasis obstruction” corresponds to neurovascular inflammation and impaired waste clearance within the glymphatic and vascular systems (10). Thus, the TCM framework can be reinterpreted as a multi-system imbalance encompassing neurotransmitter dysfunction, neuroinflammation, oxidative stress, and disturbed neurovascular coupling (11). This conceptual alignment underscores the idea that acupuncture—rooted in restoring systemic homeostasis—may exert therapeutic effects through multi-level neural regulation (12). Integrating this traditional systems-based view with modern mechanistic evidence provides a coherent rationale for exploring acupuncture’s role in promoting neuroplasticity and cognitive recovery in PSCI.

However, previous studies have typically focused on isolated molecular or regional mechanisms—for example, single neurotransmitters, inflammatory cytokines, or individual brain regions—without clarifying how these diverse pathways interact within a systems framework (13). As a result, the neurobiological basis of acupuncture for PSCI remains fragmented, and the field lacks a coherent model that links cellular processes to higher-order cognitive recovery (14).

Therefore, the present review aims to establish an integrative neurobiological framework of acupuncture in PSCI, synthesizing evidence across molecular, cellular, and network levels. We summarize current clinical and experimental findings within five interrelated dimensions: regulation of neurotransmitters and synaptic plasticity, immune-inflammatory modulation, anti-oxidative and anti-apoptotic protection, activation of BDNF-related signaling, and reconstruction of functional brain networks. By tracing the continuum from neurotransmission to brain network remodeling, this review seeks to decode how acupuncture orchestrates multi-level neurorepair and to identify future directions for precision-based acupuncture rehabilitation in cognitive recovery after stroke.

## 2 Methods

This review was conducted following the Preferred Reporting Items for Systematic Reviews and Meta-Analyses Extension for Scoping Reviews (PRISMA-ScR) guidelines. A systematic literature search was performed in PubMed, Web of Science, and Embase databases covering the period from January 2001 to February 2025, with the final search conducted on June 30, 2025. The following Boolean search strategy was applied: (acupuncture OR electroacupuncture OR manual acupuncture) AND (post-stroke cognitive impairment OR vascular cognitive impairment OR poststroke dementia OR PSCI) AND (mechanism OR neuroplasticity OR neurotransmitter OR BDNF OR inflammation OR oxidative stress OR network). The complete search strategies for each database, including search fields and limits, are provided in Supplementary Table S1. Retrieved records were imported into EndNote X9 for deduplication. Duplicate records were identified and removed automatically and manually verified. Two reviewers independently screened titles, abstracts, and full texts. Formal methodological quality assessment or risk-of-bias evaluation was not performed, as this review aimed to synthesize mechanistic evidence across heterogeneous experimental and clinical study designs rather than to quantitatively compare intervention effects. Inclusion criteria were: (1) clinical or experimental studies investigating the mechanisms of acupuncture in PSCI; (2) studies reporting molecular, cellular, or systems-level outcomes related to cognition; (3) peer-reviewed English-language publications. Exclusion criteria were: (1) conference abstracts, reviews, or case reports; (2) studies lacking mechanistic outcome measures; (3) duplicate publications.

**Figure 1:**
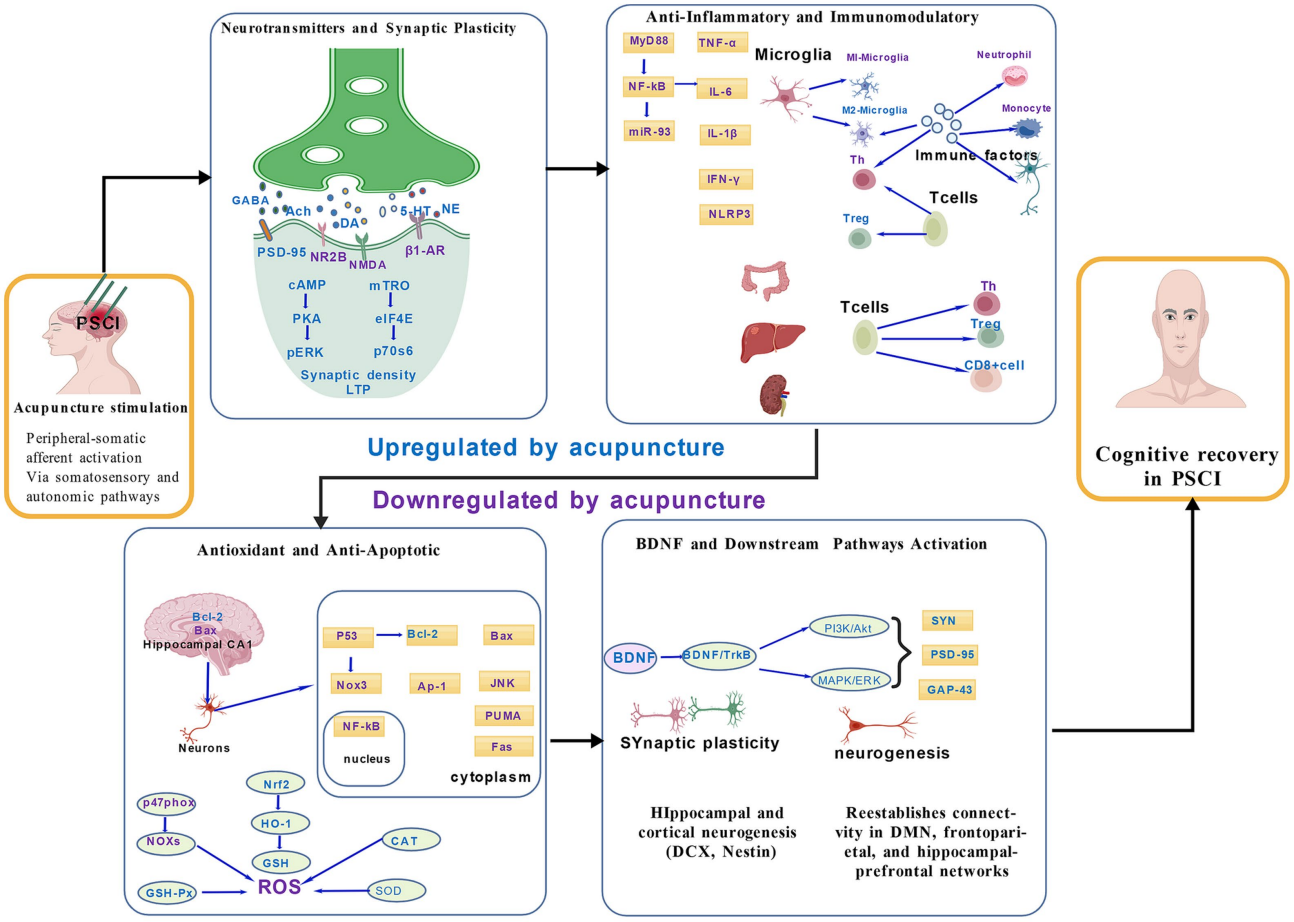
This figure presents an evidence-informed integrative framework linking peripheral-to-central signaling with multi-level neuroplastic mechanisms underlying acupuncture-induced cognitive modulation. Acupuncture initiates peripheral somatosensory and autonomic afferent activation, which is transmitted through representative spinal and brainstem relay structures to higher-order limbic and cortical regions and large-scale cognitive networks, as supported by convergent experimental, neurochemical, and neuroimaging evidence. At the molecular and cellular levels, acupuncture triggers multi-level cascades involving neurotransmitter regulation, neuroimmune modulation, antioxidative and anti-apoptotic protection, and BDNF-related neurotrophic signaling. These processes converge to promote neurogenesis and large-scale brain network remodeling, ultimately contributing to cognitive restoration in PSCI. The diagram highlights hierarchical interactions from molecular to systems levels, emphasizing acupuncture’s role as a multi-target neuromodulatory intervention. Factors in blue are upregulated by acupuncture, while factors in purple are down-regulated by acupuncture. IS, Ischemic Stroke; ICH, Intracerebral Hemorrhage; NMDA, N-methyl-d-aspartate receptor; cAMP, Cyclic Adenosine Monophosphate; PKA, Protein Kinase A; pERK, Phospho-Extracellular Signal-Regulated Kinase; BDNF, Brain-Derived Neurotrophic Factor; MyD88, Bone Marrow Differentiation Factor 88; IL-1*β*, Interleukin-1β; IFN-*γ*, Interferon-gamma; Bcl-2, B-Cell Lymphoma 2; SOD, Superoxide Dismutase. Created with BioGDP.com.

A total of 61 articles met the inclusion criteria, including 41 basic studies and 20 clinical studies (see Figure 1 for PRISMA flow diagram). Data were extracted for study type, model or patient characteristics, intervention protocol, outcome indicators, and mechanistic findings. Discrepancies were resolved by consensus. Details are provided in Tables 1, 2.

**Table 1:** Clinical effects of acupuncture treatment for PSCI.

References	Intervention method	Study design	Sample size (n)	Stroke stage	Acupoints	Acupuncture parameters	Effect measures	Outcomes	Adverse events
Dandan et al. (49)	EA	randomized controlled trial	*n* = 210	Subacute (3–6 months)	GB20 + GV25 + LI20	30 min seventimes/week4 weeks	MoCA MMSE	MoCA↑ MMSE↑	None reported
Zhong et al. (50)	SA	randomized controlled trial	*n* = 54	Subacute (3–6 months)	GB20 + GV20 + GB6	30 min sixtimes/week 4 weeks	MoCA MMSE	MoCA↑ MMSE↑	None reported
Jiao et al. (51)	SA	randomized controlled trial	*n* = 263	not clearly defined	DU20 + DU24 + EX-HN3	30 min sixtimes/week 8 weeks	MoCA MMSE	MoCA↑ MMSE↑	None reported
Zhang et al. (52)	IDSA	randomized controlled trial	*n* = 660	Subacute (3–6 months)	MS6 + GV21 + GB6 + MS7	30 min sixtimes/week 8 weeks	MMSE MoCA HAMD PSQI	MMSE↑ MoCA↑ HAMD↑ PSQI↑	None reported
Zhi et al. (53)	MA	controlled trial	*n* = 60	not clearly defined	CV17 + CV12 + CV6 + SP10	20 min sixtimes/week 12 weeks	CD3 + T CD4 + T IFN-γ TNF-α	CD3 + T↑ CD4 + T↑ IFN-γ↑ TNF-*α*↓	None reported
Wang et al. (54)	MA	Clinical Trial	*n* = 37	not clearly defined	Scalp acupuncture	30 min, sixtimes/week 4 weeks	MoCA MMSE ADL	MoCA↑ MMSE↑ ADL↑	None reported
Yang et al. (36)	MA	randomized controlled trial	*n* = 216	not clearly defined	ST36 + SP10 + CV17 + CV12	30 min sixtimes/week 12 weeks	ADAS-cog CDT ADL	ADAS-cog↑ CDT↑ ADL↑	None reported
Shi et al. (55)	MA	randomized controlled trial	*n* = 68	>2 months	GV20 + GV24 + EX-HN1 + CV17	30 min sixtimes/week 6 weeks	MMSE ADL DEMQOL	MMSE↑ ADL↑ DEMQOL↑	None reported
Shi et al. (56)	MA	randomized controlled trial	*n* = 63	Subacute (3–6 months)	GV20 + EX-HN1 + GV24 + CV17	30 min sixtimes/week 6 weeks	SDSVD	SDSVD↑	None reported
Feng (57)	EA	randomized controlled trial	*n* = 72	Subacute (3–6 months)	GV24 + GB13 + EX-B2	15 min fivetimes/week 4 weeks	ADL	ADL↑	None reported
Shi et al. (58)	MA	randomized controlled trial	*n* = 16	Subacute (3–6 months)	GV20 + GV24 + EX-HN1 + PC6	15 min sixtimes/week 6 weeks	MMSE ADL DEMQOLSDSVD	MMSE↑ ADL↑ DEMQOL↑ SDSVD↑	None reported
Chou et al. (59)	EA	randomized controlled trial	*n* = 33	not clearly defined	PC6 + HT7	30 min sixtimes/week 8 weeks	LOTCA SF-36 SS-QOL	LOTCA↑ SF-36↑ SS-QOL↑	None reported
Zhao et al. (60)	EA	randomized controlled trial	*n* = 90	not clearly defined	GV20 + GV24 + EX-HN1	30 min fivetimes/week 6 weeks	MMSE	MMSE↑	None reported
Huang et al. (61); Huang et al. (62)	MA	Clinical Trial	*n* = 72	not clearly defined	LI15 + SP10 + SJ5 + LI4	20 min fivetimes/week 4 weeks	Glucose metabolism	Glucose metabolism↑	None reported
Huang et al. (61); Huang et al. (62)	MA	randomized controlled trial	*n* = 50	Subacute (3–6 months)	GV20 + GV26 + HT7	20 min fivetimes/week 4 weeks	MMSE ADL FAQ	MMSE↑ ADL↑ FAQ↑	None reported
Yu et al. (63)	MA	randomized controlled trial	*n* = 60	not clearly defined	CV17 + CV12 + CV6 + ST36	30 min seventimes/week6 weeks	MMSE HDS-R ADL	MMSE↑ HDS-R↑ ADL↑	None reported
Lai & Huang (64)	MA	randomized controlled trial	*n* = 50	Subacute (3–6 months)	LI15 + LI11 + SJ5	20 min fivetimes/week 4 weeks	HDS-R ADL FAQ	HDS-R↑ ADL↑ FAQ↑	None reported
Liu (65)	MA	Clinical Trial	*n* = 42	not clearly defined	GV20 + GV24 + GB13	30 min fivetimes/week 8 weeks	TC + TG + LDL ApoA1 ApoB100	TC + TG + LDL↓ ApoA1↑ ApoB100↓	None reported
Chen (66)	MA	Clinical Trial	*n* = 32	not clearly defined	GV20 + GV16 + GV26 + LI11	30 min fiveimes/week 8 weeks	CCSE FAQ	CCSE↓ FAQ↓	None reported
Gao et al. (67)	MA	randomized controlled trial	*n* = 63	not clearly defined	GV26 + PC6 + SP6	15 min 5times/week 8 weeks	HDS P300 SOD LPO	HDS↑ P300↓ SOD↑ LPO↓	None reported

↑, upregulated by acupuncture; ↓, downregulated by acupuncture.

**Table 2:** Effects and mechanisms of acupuncture treating PSCI.

References	Models	Intervention methods	Acupoints	Acupuncture parameters	Effect measures	Biochemical measurements
Zhang et al. (15)	MCAO/R	EA	**-**	30 min fiveimes/week 2 weeks	Zea-Longa neurological deficitscore OFT NORT	IL-33↑ TNF-α↓ IL-1β↓ IL-6↓ M2microglial polarization↑
Chen et al. (16)	MCAO/R	EA	GV24 + GV20	30 min Sixtimes/week 2 weeks	SIRT1 PGC-1α OPA1 DRP1	SIRT1↑ PGC-1α↑ OPA1↑ DRP1 ↓
Zhang et al. (12)	MCAO	EA	DU20 + EX-HN3	20 min Sixtimes/week 4 weeks	MWM GluA1Syp Syt-1	GluA1↑ Syp↑ Syt-1 ↑
Sa et al. (21)	MCAO	EA	GV24 + GV20	1 weeks	Neurological deficit	Hspb1↑
Qiuping et al. (22)	BCCAO	EA	CV17 + CV12 + ST36	30 min fivetimes/week 1 weeks	MWM	Th17 cells↓ Treg cell↑ RORcells↓ CD4FoxP3cells↑ FoxP3 cells↑
Liu et al. (4), Liu et al. (5)	BCCAO	EA	GV20 + BL17 + BL23	30 min fivetimes/week 1 weeks	MWM	Bcl-2↑ AP-1↓ p53↓ JNK↓ Caspase-3↓
Zhao et al. (23)	BCCAO	EA	ST36 + GV20	-	MWM	Sirt1↑ STAT3↓ IL-17↓
Bu et al. (42)	4VO	EA	GV20 + CV17 + BL17	30 min fivetimes/week 3 weeks	MWM	IL-6↓ TNF-α↓ TLR4↓ MyD88↓
Dai et al. (24)	BCCAO	EA	GV20 + GV24	30 min fivetimes/week 4 weeks	NORT MWM	p-NMDAR2B p-GluR1↑ p-CaMKII↑
Ma et al. (38)	BCCAO	EA	GV20 + GV24	30 min fivetimes/week 3 weeks	MWM	miR-81↑ IL-16↓ PSD-95↑
Pan et al. (13)	BCCAO	EA	CV17 + CV12 + CV6 + ST36	30 min fivetimes/week 3 weeks	MWM	IL-1β↓ IL-2↓ TNF-α↓ COX-2↓ IL-4↑
Wang et al. (11)	MCAO	EA	GV20 + GV24	30 min sixtimes/week 1 weeks	MWM	Beclin-1↑ mTOR↑ PI3K↑ p-Akt↑ p-Beclin-1↑ p-PI3K↑
Wang et al. (11)	MCAO	EA	GV2 + BL17 + BL20 + BL23	15 min sixtimes/week 2 weeks	MWM	AVP↑ SS↑ β-EP↑
Ma et al. (33)	BCCAO	MA	GV20 + ST36	15 min sixtimes/week 2 weeks	NORT, MWM	CBF↑ IL-1β↓ IL-6↓
Wang et al. (68), Wang et al. (54)	BCCAO	MA	ST36 + GV20	15 min sixtimes/week 2 weeks	MWM	IL-6↓ TNF-α↓ TLR4↓ MyD88↓ p-NF-κB p65↓
Yang et al. (34)	BCCAO	MA	ST36 + GV20	15 min fivetimes/week 2 weeks	MWM	Neuronal damage↑
Yang et al. (36), Yang et al. (37)	CMi	MA	ST36	30 min sixtimes/week 3 weeks	MWM	NF-kB↓ NF-kB p65 p53↓ Ca2+, ROS↓
Zhu et al. (14)	BCCAO	MA	GV20 + ST36	30 min fivetimes/week 2 weeks	MWM	Trx-1↓ TrxR-1↓ pASK1↓ pJNK↓ p38↓
Du et al. (69)	BCCAO	MA	GV20 + ST36	15 min fivetimes/week 4 weeks	MWM	TXNIP↓
Yang et al. (10)	BCCAO	EA	ST36 + GV20	15 min fivetimes/week 2 weeks	MWM	ROS↓ LTP↑
Xiao et al. (70)	BCCAO	MA	ST36 + GV20	30 min sixtimes/week 4 weeks	MWM	LTP↑β1-AR↑ NE↑
Ye et al. (35)	BCCAO	MA	ST36 + GV20	30 min sixtimes/week 2 weeks	MWM	DA↑ epinephrine↑ HVA↑ D1R↑ D5R↑
Li et al. (32)	BCCAO	MA	GV20 + ST36	15 min sixtimes/week 3 weeks	MWM	Mitochondrial respiratory complexenzymes (complex I, II, IV)↑ cytochrome coxidase IV↑ ROS↓
Yang et al. (71)	BCCAO	EA	GV20 + KI3	30 min fivetimes/week 3 weeks	Y-Maze Task	Iba-1↓ TLR4↓ TNF-α↓ pERK↑ glucosemetabolism↑
Zhang et al. (39)	MCAO	EA	GV20 + GV24	30 min sixtimes/week 1 weeks	Step-down avoidance test	CaM↓ CaMKIV↑ p-CaMKIV↑ CREB↑ p-CREB↑
Liu et al. (40)	MCAO	EA	GV20 + GV24	15 min sixtimes/week 2 weeks	Neurological deficitscores, MWM	Bcl-2↑ Bax↓ neuronal apoptosis↓
Li et al. (84)	CMi	MA	ST36	20 min sixtimes/week 4 weeks	MWM	Pyramidal neurons↑ astrocytes↓
Li et al. (48)	CMi	MA	ST36	15 min fivetimes/week 2 weeks	MWM	cAMP concentration↑ PKAactivity↑ pCREB↑ pERK↑
Yang et al. (72)	BCCAO	MA	GV2 + GV21 + GV22 + GV24	15 min sixtimes/week 4 weeks	MWM	ACh↑ DA↑ 5-HT↑
Zhang et al. (41)	CMi	MA	CV17 + CV12 + CV6 + ST36	30 min sixtimes/week 3 weeks	MWM	CBF↑ SOD↑ CuZnSOD↑ MnSOD↑ MDA↓
Zhu et al. (73)	BCCAO	EA	GV20 + GV14 + BL23	20 min sixtimes/week 4 weeks	MWM	mTOR↑ eIF4E↑
Feng et al. (74)	MCAO	EA	GV20 + GV24	15 min sixtimes/week 1 weeks	MWM	NF-κB↓ neurons↑
Zhu et al. (75)	BCCAO	EA	GV20 + GV14 + BL23	20 min sixtimes/week 4 weeks	MWM	p70S6↑ ribosome protein S6↑
He (76)	4VO	EA	HT9 + PC9 + KI1	30 min sixtimes/week 2 weeks	MWM	NO↓ SOD↑
Zhao et al. (77)	CMi	MA	CV17 + CV12 + CV6 + ST36	20 min sixtimes/week 3 weeks	MWM	Hexokinase↑ pyruvate kinase↑ glucose6phosphatedehydrogenase↑
Zhu & Zeng (78)	BCCAO	EA	GV20 + GV24 + BL23	20 min sixtimes/week 2 weeks	MWM	Pyramidal neurons↑ p53↓ Noxa↓
Lin & Hsieh (79)	MCAO	EA	GV20	20 min fivetimes/week 2 weeks	Behavior deficit score(18-point scale)	LTP↑ NMDAR1↓ TRPV1↓
Wang et al. (80)	CMi	MA	CV17 + CV12 + CV6 + ST36	20 min sixtimes/week 2 weeks	MWM	Bcl-2↑ Bax↓ neuronal apoptosis↓
Zhu et al. (81)	BCCAO	EA	GV20 + GV24 + BL23	20 min sixtimes/week 4 weeks	MWM	Neurons↑ Caspase-3↓ Noxa↓
Yu et al. (82)	CMi	MA	CV17 + CV12 + CV6 + ST36	15 min sixtimes/week 3 weeks	MWM	Escape latency↓
Wang et al. (83)	4VO	EA	GV20 + GV14	20 min fivetimes/week 2 weeks	MWM	NO↓ MDA↓ NOS↓ SOD↑ GSH-Px↑

↑, upregulated by acupuncture; ↓, downregulated by acupuncture. This table summarizes mechanistic evidence derived from experimental models relevant to post-stroke cognitive impairment (PSCI), including BCCAO, 4VO, CMi, and MCAO/R models. These models represent distinct pathological trajectories and temporal profiles of neuroinflammation and neurodegeneration. IDSA, interactive dynamic scalp acupuncture; MCAO, middle cerebral artery occlusion; MCAO/R, MCAO with reperfusion; BCCAO, bilateral common carotid artery occlusion; 4VO, modified 4-vessel occlusion; CMi, cerebral multi-infarction; GB20, Fengchi; GV24, Shenting; LI20Ying xiang; GV20, Baihui; GB6, xuanli; DU20, Baihui; EX-HN3, yintangGV14, Dazhui; GV16, FengfuBL11dazhu; MS6, dingnieqianxiexian; GV21, Qianding; MS7, dingniehouxiexian; CV17, Danzhong; CV12, zhongwan; CV6, Qihai; SP10, Xuehai; ST36, Zu San Li; EX-HN1, sishencon; PC6, neiguan; GB13, Benshen; EX-B2, jiaji; HT7, Shenmen; LI15, Jianyu; SJ5, waiguan; LI4, Hegu; SP6, SanYinJiao; LR3 TaiChon; GV26, Shuigou; LI11, Quchi; BL17, Geshu; BL23, Shenshu; KI3, Taixi; GV22, Xinhui; HT9, shaochong; PC9 Zhong chong; KL1, Yongquan; BL20, Pishu; SDSVD, scale of differentiation of syndromes of vascular dementia; HAMD, Hamilton Depression Scale; PSQI, Pittsburgh Sleep Quality Index; ADL, Activities of Daily Living Scale; SDS, Self-rating Depression Scale; SDSVD, scale of differentiation of syndromes of vascular dementia; FAQ, Functional activities Questionnaire; HDS-R, Hasegawa’s dementia scale; SF-36, 36-item short-form health survey; SS-QOL, Stroke-Specific Quality of Life Scale; SOD, suoeroxide dismutase; LOP, lipidperoxide; NDS, neurologic deficit soring; IS, ischemicstroke; ICH, intracerebralhemorrhage; TEAS, transcutaneous acupoint electrical stimulation; Bcl-2, B-cell lymphoma 2; NF-κB, nuclear factors-κB; PSD-95, postsynaptic density protein-95; LTP, long-term potentiation; LTD, long-term depression; NE, norepinephrine; AR, adrenergic receptor; TRPV1, transient receptor potential vanilloid subtype 1; NMDA, N-methyl-d-aspartate; CMi, cerebral multi-infarction; NMDAR, N-methyl-d-aspartate receptor; CaM, calmodulin; CaMKIV, calmodulin-dependent protein kinase type IV; CREB, cyclic adenosine monophosphateresponse elements binding protein; AMPARs, α-amino-3-hydroxy-5-methyl-4-isoxazolepropionic acid receptors; mTOR, mammalian target of rapamycin; p70S6, p70 ribosomal protein S6; eIF4E, eukaryotic translation initiation factor 4E; DA, dopamine; HVA, homovanillic acid; PP, perforant pathway; DG, dentategyrus; Ach, acetylcholine; 5-HT, 5-hydroxytryptamine; TLRs, Toll like receptors; PAMPs, pathogen associated molecule pattern; DAMPs, damage associated molecular patterns; MyD88, bonemarrow differentiation factor 88; Hspb1, heat-shock proteinβ1; pERK, phospho-extracellular signal-regulated kinase; Sirt1, sirtuin1; STAT3, signal transducer and activator of transcription3; CBF, cerebral bloodflow; DTI, diffusion tensor imaging; RNS, reactive nitrogen; ROS, reactive oxygen species; NO, nitric oxide; MDA, malondialdehyde; NOS, NO synthase; GSH-Px, glutathione peroxidase; MID, multi-infarct dementia; GSSG, oxidized glutathione; RCI, respiratory control index; TXNIP, thioredoxin-interacting protein; Trx, thioredoxin.

## 3 Mechanistic advances in acupuncture for PSCI

### 3.1 Regulation of neurotransmitters and synaptic plasticity

Acupuncture modulates the balance of excitatory and inhibitory neurotransmission disrupted after stroke, serving as the initial molecular trigger of a multi-level neurorepair cascade. The maintenance of cognitive function relies heavily on intact synaptic connectivity and the efficient transmission of information between neurons within neural circuits (15). Homeostatic regulation of neurotransmitter systems and dynamic modulation of synaptic plasticity are essential for higher-order cognitive processes, including attention, learning, and memory. Following stroke, disturbances in neurotransmitter signaling and structural damage to synapses represent key mechanisms underlying PSCI (16). These changes disrupt excitatory-inhibitory balance, impair synaptic integration, and reduce cognitive reserve. A growing body of evidence indicates that acupuncture can restore neurotransmitter homeostasis and enhance synaptic plasticity via multiple molecular pathways, ultimately contributing to improved cognitive outcomes after stroke (12). ACh is a key neurotransmitter in the central nervous system, primarily synthesized and released by cholinergic neurons. These neurons are predominantly located in the basal forebrain and project to critical cognitive regions such as the hippocampus, neocortex, and brainstem (17). Through extensive synaptic connections, cholinergic circuits support essential cognitive processes, including memory encoding, learning, and attention regulation. ACh plays a central role in maintaining cortical arousal and attentional modulation via the ascending reticular activating system (ARAS) of the brainstem. Disruption of ACh signaling—particularly in the cortex and hippocampus—has been implicated in deficits in spatial learning, memory consolidation, and overall cognitive decline following stroke (8). DA, a catecholaminergic neurotransmitter, plays a critical role in regulating cognition, particularly in domains such as motivation, working memory, and executive function. Stroke-induced degeneration of dopaminergic neurons, particularly in the mesocortical and mesolimbic pathways, has been linked to deficits in attention, executive processing, and reward-based learning (18). NE, predominantly released by neurons in the locus coeruleus, contributes to the modulation of arousal, attention, learning, and memory consolidation. Disruption of noradrenergic pathways following stroke may impair cortical activation and reduce the brain’s capacity for attentional control, thereby exacerbating cognitive impairment (19). 5-HTsynthesized in the raphe nuclei, is another key monoaminergic neurotransmitter that regulates not only mood and emotional stability, but also spatial learning, memory encoding, and circadian rhythm (20). Ischemic damage to serotonergic projections has been shown to impair synaptic plasticity and neurogenesis, thereby contributing to both cognitive and affective dysfunction in stroke survivors. Acupuncture has been shown to significantly modulate central neurotransmitter levels, particularly those implicated in cognitive processes such as learning, memory, and attention. Preclinical studies have demonstrated that acupuncture stimulation at specific acupoints, including GV20and GV24, increases ACh levels in the hippocampus and cortex, enhances cholinergic neurotransmission, and ameliorates memory deficits and attentional impairments following stroke. Moreover, acupuncture has been reported to downregulate acetylcholinesterase (AChE) activity, thereby reducing ACh degradation and promoting cholinergic homeostasis. This balance between ACh synthesis and degradation is crucial for maintaining synaptic plasticity and cognitive integrity, and its restoration through acupuncture contributes to improved learning and memory performance in post-stroke models (9). Synaptic plasticity impairment is considered one of the major causes of cognitive dysfunction following stroke. Neural plasticity encompasses synaptic plasticity, neurogenesis, and the remodeling of axons and dendrites, reflecting the brain’s adaptive response to injury and environmental changes (21). Impaired neuroplasticity can lead to persistent cognitive deficits in patients. In Alzheimer’s disease, structural synaptic alterations such as synaptic loss and reduced synaptic density occur at an early stage (22). In patients with vascular dementia, inadequate cerebral perfusion, accumulation of reactive oxygen species and lactic acid, and inflammatory responses may lead to reduced synapse number, smaller active zones of synaptic connections, and neuronal cell death. Impaired synaptic plasticity is widely recognized as a fundamental contributor to cognitive deficits following stroke (23). Neural plasticity encompasses synaptic remodeling, neurogenesis, and structural modifications of axons and dendrites, reflecting the brain’s intrinsic capacity to adapt to injury and environmental stimuli (24). Disruption of neuroplastic processes may result in long-term impairments in cognitive flexibility, learning capacity, and memory consolidation. In Alzheimer’s disease, structural synaptic alterations—including synaptic loss and reduced synaptic density—emerge early and are strongly associated with cognitive decline. Similarly, in vascular dementia, chronic cerebral hypoperfusion, oxidative stress, and neuroinflammation contribute to synapse loss, shrinkage of active synaptic zones, and progressive neuronal degeneration (4, 5). Ischemic stroke induces microstructural alterations in neural networks, including thickening of postsynaptic density (PSD), reduced extracellular space, decreased dendritic spine density, and diminished mitochondrial number within synapses—factors that collectively impair energy metabolism and hinder efficient synaptic transmission, thereby contributing to PSCI (13). Studies have shown that acupuncture promotes the expression of synapse-associated proteins and facilitates the restoration of synaptic structure and function. Specifically, acupuncture has been shown to upregulate hippocampal expression of key synaptic markers, including synaptophysin (SYN) and PSD-95, thereby enhancing synaptic density, maturation, and structural remodeling. Moreover, acupuncture enhancesLTP in the hippocampus, thereby strengthening synaptic transmission efficiency and providing a physiological basis for improved learning and memory functions (11).

In summary, acupuncture contributes significantly to the reconstruction of post-stroke neural networks through bidirectional modulation of neurotransmitter systems and enhancement of synaptic plasticity. These effects constitute a fundamental mechanism by which acupuncture improves cognitive outcomes in patients with PSCI. By restoring synaptic efficiency and reactivating cholinergic and dopaminergic signaling, acupuncture establishes a neurochemical foundation that subsequently influences neuroinflammatory and neuroimmune responses.

### 3.2 Anti-inflammatory and immunomodulatory mechanisms

Following the rebalancing of neurotransmission, acupuncture exerts potent regulatory effects on neuroinflammation and immune crosstalk within the injured brain. Neuroinflammation is recognized as a central pathogenic mechanism underlying PSCI. Stroke triggers the activation of the central immune system, resulting in overactivation of microglia and the subsequent release of pro-inflammatory cytokines, including interleukin-1β (IL-1β), tumor necrosis factor-*α* (TNF-α), and interleukin-6 (IL-6). These cytokines further promote the recruitment of peripheral immune cells—such as monocytes, lymphocytes, and neutrophils—into the ischemic brain tissue (25). The infiltration of inflammatory cytokines and peripheral immune cells exacerbates neuronal damage, impairs synaptic integrity, and disrupts functional neural circuits, thereby accelerating cognitive deterioration. In recent years, accumulating evidence has demonstrated that acupuncture can suppress the activation, infiltration, and proliferation of inflammatory cells in the central nervous system (26). It also helps restore the balance between pro-inflammatory and anti-inflammatory mediators, thereby exerting significant neuroprotective effects in ischemic brain injury. Animal studies have confirmed that acupuncture stimulation at specific acupoints—such as GV20 and Dazhui (GV14)—significantly downregulates the expression of pro-inflammatory cytokines, including IL-1β, TNF-*α*, and others in brain tissue. In addition, acupuncture inhibits the activity of inflammation-related pathways, particularly the Toll-like receptor 4 (TLR4)/NF-κB signaling cascade, thereby attenuating neuroinflammatory responses. NF-κB, a canonical inflammatory signaling mediator, upon activation induces the transcription of multiple pro-inflammatory cytokines and perpetuates the inflammatory cascade (2). Acupuncture has been shown to inhibit NF-κB phosphorylation and nuclear translocation, thereby suppressing its transcriptional activity and reducing cytokine production at the upstream regulatory level. Microglia, the resident macrophages of the central nervous system, play a dual role in neuroinflammation and can induce neuronal degeneration and death under pathological conditions (1). Acupuncture has been shown to modulate microglial polarization by promoting the phenotypic shift from the pro-inflammatory M1 type to the anti-inflammatory M2 type (27). This shift enhances the secretion of neuroprotective cytokines such as interleukin-10 (IL-10) and transforming growth factor-*β* (TGF-β), while suppressing the pro-inflammatory cytokine cascade, thereby creating a neuroimmune microenvironment conducive to neural repair. Furthermore, acupuncture has been reported to inhibit inflammasome activation, particularly the NOD-like receptor protein 3 (NLRP3) inflammasome, thereby reducing neuronal pyroptosis and preserving the structural integrity of brain tissue after stroke. In addition to its central effects, acupuncture also modulates peripheral immune responses via the neuroimmune axis (28). It has been demonstrated that acupuncture activates the vagus nerve-mediated cholinergic anti-inflammatory pathway, which suppresses the production of inflammatory cytokines in the spleen and peripheral immune organs. These findings highlight the systemic anti-inflammatory effects of acupuncture (29).

In summary, acupuncture alleviates chronic neuroinflammation following stroke through multi-level, multi-target anti-inflammatory mechanisms. These include the suppression of pro-inflammatory cytokine expression, modulation of key signaling pathways, and regulation of glial activation and immune cell polarization. Collectively, these mechanisms attenuate sustained neuroinflammatory damage and establish an immunological foundation for cognitive recovery in post-stroke patients. These anti-inflammatory and immune-modulatory effects not only mitigate secondary neuronal damage but also reduce oxidative burden, paving the way for the activation of antioxidant and anti-apoptotic pathways.

### 3.3 Antioxidant and anti-apoptotic mechanisms

As inflammation subsides, oxidative stress and apoptosis remain key barriers to neural survival and network integrity; acupuncture has been shown to alleviate these processes through multiple signaling pathways. Oxidative stress, caused by an imbalance between pro-oxidant and antioxidant systems, is recognized as a central pathological mechanism contributing to PSCI (6, 7). During cerebral ischemia–reperfusion injury, excessive production of ROS initiates a cascade of oxidative stress responses (3). These reactive species damage membrane lipids, proteins, and nucleic acids, impair mitochondrial function, and ultimately induce neuronal apoptosis and necrosis, all of which contribute significantly to cognitive dysfunction. In addition, oxidative stress indirectly exacerbates neuronal injury by disrupting intracellular signaling pathways, impairing energy metabolism, and further aggravating mitochondrial dysfunction. ROS also disrupt the integrity of the blood–brain barrier (BBB) by damaging endothelial cells, increasing permeability, and facilitating the infiltration of inflammatory cells and cytokines into brain parenchyma, thereby exacerbating tissue injury (30). Sustained oxidative stress may initiate chronic neuroinflammation, characterized by persistent microglial activation, excessive ROS generation, and upregulation of pro-inflammatory cytokines, forming a vicious cycle that further damages neurons and impairs neuroregeneration (31). Recent studies suggest that acupuncture exerts neuroprotective and cognition-enhancing effects by upregulating endogenous antioxidant defenses and modulating apoptosis-related signaling cascades (9). First, acupuncture has been shown to effectively reduce oxidative stress levels in brain tissue following stroke. Experimental studies indicate that acupuncture upregulates the activity of endogenous antioxidant enzymes, including superoxide dismutase (SOD), while concurrently downregulating lipid peroxidation markers such as malondialdehyde (MDA) (20). These effects alleviate free radical-induced cellular damage and contribute to the stabilization of the neural microenvironment. In addition, acupuncture enhances other antioxidant defense systems, including GSH-Px and catalase (CAT), thereby further augmenting the brain’s capacity to counteract oxidative damage (4, 5). Apoptosis, also referred to as programmed cell death, is a genetically regulated and highly orchestrated process of cellular self-destruction (6, 7). It plays essential roles in embryonic development, tissue remodeling, and immune homeostasis. Neuronal apoptosis is a core pathological event in the development of PSCI (32). During cerebral ischemia and hypoxia, cellular energy metabolism collapses, ATP synthesis declines sharply, and membrane ion pumps become dysfunctional, leading to intracellular calcium overload and a cascade of cytotoxic events that culminate in neuronal apoptosis. The Wnt/*β*-catenin signaling pathway plays a pivotal role in promoting neuronal survival and inhibiting apoptosis (33). Under physiological conditions, stabilized β-catenin translocates to the nucleus, where it interacts with TCF/LEF transcription factors to upregulate anti-apoptotic genes such as Bcl-2 and suppress pro-apoptotic proteins including Bax and caspase-3, thereby preventing neuronal apoptosis. In PSCI, ischemia and hypoxia lead to abnormal activation of GSK-3*β*, which induces excessive phosphorylation and proteasomal degradation of β-catenin, thereby markedly reducing its cytoplasmic levels (34). This diminishes anti-apoptotic capacity, promotes cytochrome c release, and activates the caspase cascade, culminating in neuronal apoptosis. Extensive neuronal apoptosis leads to severe disruption of neural circuits, particularly in the hippocampal CA1 region and prefrontal cortex, and is considered a pathological hallmark of PSCI (14). Studies have demonstrated that acupuncture mitigates post-stroke neuronal apoptosis through multiple molecular mechanisms. It modulates the expression of Bcl-2 family proteins by upregulating anti-apoptotic Bcl-2 and downregulating pro-apoptotic Bax, inhibits mitochondrial membrane permeability transition, and prevents cytochrome c release—collectively suppressing mitochondria-dependent apoptotic pathways (35). Moreover, acupuncture downregulates the expression and enzymatic activity of caspase-3 and caspase-9—critical downstream effectors of the apoptotic cascade—thereby preserving the structural and functional integrity of brain tissue. Emerging evidence also suggests that acupuncture may regulate endoplasmic reticulum stress and modulate the crosstalk between autophagy and apoptosis following stroke, offering additional molecular targets for neuroprotection (36, 37).

In summary, acupuncture exerts coordinated neuroprotective effects through multiple mechanisms, including enhancement of antioxidant defenses, suppression of free radical accumulation, and modulation of mitochondrial pathways and downstream apoptotic executioners. These mechanisms collectively mitigate oxidative damage and programmed neuronal death following stroke, thereby establishing a crucial cellular foundation for cognitive recovery. The attenuation of oxidative injury and apoptosis creates a favorable intracellular environment for neurotrophic signaling, particularly the up-regulation of BDNF and its downstream cascades.

### 3.4 Regulation of BDNF and its downstream signaling pathways

Building upon the neuroprotective microenvironment established by antioxidant mechanisms, acupuncture activates neurotrophic signaling centered on the brain-derived neurotrophic factor (BDNF) pathway. BDNF is the most abundant neurotrophin in the central nervous system. It plays a pivotal role in neuronal survival, synaptic plasticity, and the regulation of learning and memory (38). BDNF also contributes to neuronal repair following injury and prevents degenerative changes in neural cells, with its highest concentrations found in the hippocampus and cortex. BDNF exerts its biological effects by binding to its high-affinity receptor, tropomyosin receptor kinase B (TrkB), inducing tyrosine phosphorylation and activating downstream signaling cascades (39). The BDNF/TrkB signaling pathway is essential for neuronal survival and proliferation, dendritic and axonal development, and modulation of synaptic plasticity (40). Stroke markedly downregulates BDNF expression and impairs TrkB receptor activity, leading to disrupted synaptic remodeling and diminished neuronal repair capacity. This process represents a key pathological mechanism in the development of PSCI (41). In recent years, the regulatory effect of acupuncture on BDNF and its downstream signaling pathways has gained increasing attention as a potential mechanism underlying its therapeutic effects on PSCI (6, 7). Multiple animal studies have demonstrated that acupuncture significantly upregulates BDNF mRNA and protein expression in post-stroke brain tissue, restores its binding activity with TrkB, and activates a series of neuroprotective downstream pathways (1). Notably, the BDNF–TrkB axis mediates the phosphorylation of cAMP response element-binding protein (CREB), which enhances the expression of plasticity-related proteins such as SYN and growth-associated protein 43 (GAP-43), thereby facilitating synaptic remodeling and memory consolidation (26).

In addition, BDNF activates the phosphoinositide 3-kinase (PI3K)/protein kinase B (Akt) pathway, which inhibits pro-apoptotic protein expression and promotes neuronal survival (21). Acupuncture has also been shown to activate the PI3K/Akt pathway in stroke models, suggesting a potential synergistic neuroprotective effect with BDNF signaling. Additionally, acupuncture modulates other signaling cascades such as MAPK/ERK, further expanding the BDNF-mediated regulatory network. BDNF also plays a pivotal role in mediating bidirectional signaling between neurons and glial cells (42). Acupuncture-induced upregulation of BDNF expression in astrocytes may represent a novel mechanism for restoring neuron–glia interactions post-stroke (4, 5). In summary, acupuncture modulates the BDNF/TrkB signaling axis and its downstream pathways, including CREB and PI3K/Akt, thereby promoting neuronal survival, facilitating synaptic plasticity, and contributing to cognitive recovery (17). This represents a core mechanism through which acupuncture ameliorates post-stroke cognitive impairment. Through these neurotrophic cascades, acupuncture promotes synaptic remodeling, axonal regeneration, and neuronal survival, thereby facilitating large-scale network reorganization and functional integration.

### 3.5 Promotion of neurogenesis and brain network reorganization

The cumulative effects of neurotransmitter regulation, anti-inflammatory modulation, antioxidative protection, and neurotrophic activation ultimately converge at the systems level, driving neurogenesis and the reconstruction of functional brain networks. After stroke, structural damage in localized brain regions combined with disrupted functional connectivity constitutes the pathological basis of cognitive impairment (8). Stroke not only induces neuronal apoptosis and synaptic disconnection but also compromises the integrity of neural networks, particularly disrupting key cognitive circuits such as the thalamocortical and hippocampal–prefrontal pathways (43). These changes severely impair the brain’s integrative and processing capacity during cognitive tasks (6, 7). Neurogenesis and brain network reorganization represent two central processes in cognitive recovery after stroke, both of which are positively regulated by acupuncture. Regarding neurogenesis, studies have demonstrated that acupuncture promotes the proliferation, differentiation, and migration of neural stem cells (NSCs) in the hippocampal dentate gyrus (DG) and subventricular zone (SVZ) (44). This facilitates the generation of new neurons, thereby providing a cellular foundation for post-stroke brain tissue repair and cognitive recovery. Electroacupuncture at acupoints such as GV20 and Shenting GV24 modulates signaling pathways including Notch and Wnt/*β*-catenin, thereby enhancing NSC activity. It also upregulates neurogenesis-related markers such as Nestin and doublecortin (DCX), indicating that acupuncture facilitates the generation and integration of newborn neurons (4, 5).

Regarding brain network reorganization, functional magnetic resonance imaging (fMRI) studies have revealed that acupuncture modulates functional connectivity among multiple brain regions (9). Specifically, it enhances synchrony within regions associated with the default mode network (DMN), including the medial prefrontal cortex (mPFC), hippocampus, and posterior cingulate cortex (PCC), suggesting a potential role for acupuncture in restoring high-order cognitive networks disrupted by stroke (45). Additional studies have shown that acupuncture promotes synaptogenesis and remyelination, enhances axonal conduction efficiency, and provides a microstructural basis for neural network functional restoration (3). Notably, acupuncture-induced neurogenesis and functional remodeling exhibit “acupoint-to-central-target” specificity. That is, its regulatory effects are not diffuse or nonspecific, but preferentially target cognition-related brain regions such as the hippocampus and prefrontal cortex (29). This may represent a distinctive mechanistic feature distinguishing acupuncture from other physical therapies. In summary, acupuncture facilitates neurogenesis and functional connectivity remodeling post-stroke, offering dual structural and functional support for brain recovery, and forming a fundamental biological basis for its therapeutic effects on PSCI. This hierarchical progression—from molecular modulation to network reorganization—illustrates the integrative neurobiological mechanism by which acupuncture may restore cognitive function in post-stroke populations.

## 4 Discussion

PSCI arises from multifactorial risk interactions involving neurovascular injury, metabolic dysfunction, and chronic neuroinflammation (45). Clinical studies have identified age, stroke severity, lesion location, white matter integrity, hypertension, and diabetes as major determinants of PSCI development and prognosis (46). These heterogeneous risk factors converge on shared biological cascades, including oxidative stress, excitotoxicity, microglial activation, and impaired neurovascular coupling, leading to synaptic loss and network disintegration (3).

This multifactorial background explains why single-target pharmacological treatments have achieved limited success in restoring cognitive function. From a systems perspective, the complexity of PSCI underscores the need for interventions capable of simultaneously modulating multiple interdependent mechanisms. Acupuncture, characterized by its multimodal regulatory actions—encompassing neurotransmission, neuroimmune modulation, oxidative protection, and brain network remodeling—may therefore provide unique therapeutic advantages (27). Understanding how acupuncture acts upon these shared pathological axes helps contextualize its neurobiological mechanisms and clarifies its potential role within the broader framework of PSCI rehabilitation.

The mechanistic evidence summarized in this review indicates that acupuncture exerts neuroprotective and neurorestorative effects through five interrelated dimensions: (1) regulation of neurotransmitters and synaptic plasticity (47). (2) modulation of inflammatory and immune responses (4, 5). (3) suppression of oxidative stress and apoptosis (27). (4) activation of BDNF-related signaling pathways (20). (5) promotion of neurogenesis and brain network remodeling (6, 7). These mechanisms are not independent but rather form an interlocking network of molecular and systemic interactions.

At the molecular level, acupuncture restores neurotransmitter balance by increasing acetylcholine, dopamine, and serotonin while suppressing glutamate excitotoxicity, thereby facilitating synaptic plasticity. It concurrently modulates immune cascades—suppressing proinflammatory cytokines (TNF-*α*, IL-1β) (15) and enhancing anti-inflammatory mediators (IL-10)—which mitigates microglial overactivation and secondary neuronal injury (48). In parallel, acupuncture strengthens antioxidative defense by upregulating SOD and Nrf2 signaling and inhibiting lipid peroxidation markers such as MDA, contributing to improved cellular survival (29).

**Figure 2:**
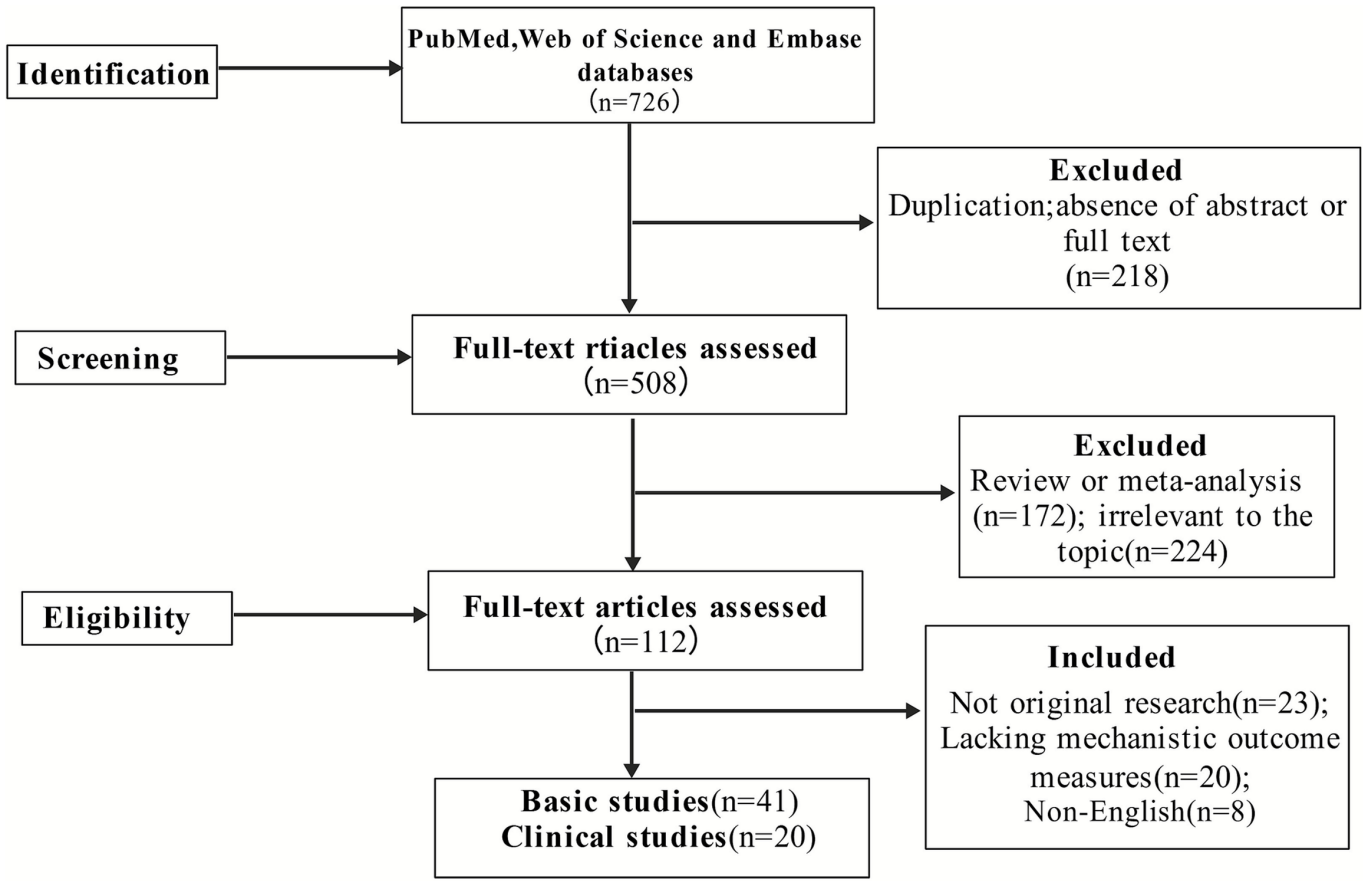
PRISMA flow diagram of literature selection (created with BioGDP.com).

Beyond these molecular and cellular effects, an important question concerns how acupuncture-related signals are transmitted and integrated across neural circuits and large-scale brain systems. Although Figure 2 is presented as an integrative schematic, the proposed peripheral-to-central afferent network is grounded in convergent experimental and clinical evidence. Peripheral acupuncture stimulation has been shown to activate somatosensory and autonomic afferents, including Aδ and C fibers as well as vagal pathways, which project to spinal and brainstem relay nuclei such as the dorsal horn, nucleus tractus solitarius (NTS), and periaqueductal gray (PAG). These subcortical structures have been repeatedly implicated in acupuncture-induced neuromodulation and are known to influence limbic and cortical regions, including the hippocampus and prefrontal cortex. Neuroimaging studies further suggest that modulation of these regions is associated with changes in large-scale cognitive networks, such as the default mode network, which are critical for post-stroke cognitive function. Accordingly, Figure 2 should be interpreted as an evidence-informed integrative framework rather than a comprehensive anatomical pathway.

At the interface between circuit-level signaling and network-level plasticity, BDNF-related pathways play a central integrative role. Furthermore, acupuncture activates the BDNF/TrkB pathway and its downstream cascades (PI3K/Akt, MAPK/ERK, CREB), which facilitate neuronal growth, axonal sprouting, and synaptic remodeling (21). These molecular and cellular effects collectively support structural reorganization within large-scale brain networks. Although an increasing number of neuroimaging studies have explored the effects of acupuncture on brain network organization after stroke, the available evidence remains limited and heterogeneous with respect to study design, post-stroke stage, imaging metrics, and cognitive endpoints. Existing resting-state fMRI studies suggest that acupuncture may modulate functional connectivity within cognition-related large-scale networks, including the default mode network (DMN), frontoparietal network, and hippocampal–prefrontal circuits. Reported network-level changes include alterations in functional connectivity strength, regional homogeneity, amplitude of low-frequency fluctuations, and graph-theoretical properties, which are broadly implicated in memory, attention, and executive processing (9). However, due to the relatively small number of PSCI-specific neuroimaging studies and substantial methodological variability across existing reports, a formal tabulated comparison of neuroimaging findings was not performed. Instead, the present review provides a narrative synthesis to contextualize neuroimaging evidence within a systems-level framework of acupuncture-induced neuroplasticity. Accordingly, current fMRI findings should be interpreted as emerging evidence supporting network-level modulation rather than definitive neuroimaging biomarkers of cognitive recovery.

The integration of molecular, cellular, and network-level evidence provides a coherent framework that aligns traditional acupuncture theory with contemporary neuroscience. From the TCM perspective, acupuncture aims to restore systemic homeostasis and harmonize interactions between the brain and viscera; in modern neurobiological terms, this corresponds to re-establishing neurotransmitter equilibrium, reducing inflammation, and promoting neurovascular and synaptic resilience. Such cross-domain correspondence highlights acupuncture as a paradigmatic “multi-target systems therapy.”

Clinically, this integrative model suggests that acupuncture’s benefits are not restricted to symptomatic relief but may involve fundamental reorganization of neural networks underlying cognition. Future clinical trials should, therefore, combine standardized cognitive assessments (MMSE, MoCA) with multimodal biomarkers—such as functional neuroimaging, electrophysiology, and molecular assays—to delineate the causal pathways between acupuncture-induced biological changes and cognitive improvement. Additionally, interventional studies that adopt precision-based approaches (e.g., personalized acupoint prescriptions guided by neuroimaging or phenotype) may enhance therapeutic specificity and reproducibility. Importantly, much of the mechanistic evidence discussed above is derived from experimental models, which warrants careful consideration of model-specific contexts. The mechanistic evidence summarized in Table 2 is derived from multiple experimental models relevant to post-stroke cognitive impairment, including chronic cerebral hypoperfusion models (e.g., BCCAO and 4VO), focal ischemia models (e.g., MCAO/R), and cerebral microinfarction models. These models differ in vascular pathology, inflammatory dynamics, and temporal progression of cognitive impairment, and therefore capture distinct facets of PSCI-related neurobiology rather than a single uniform disease process. Chronic hypoperfusion models predominantly reflect long-term white matter injury, neuroinflammatory persistence, and progressive synaptic dysfunction, which resemble the gradual cognitive decline observed in many PSCI patients. In contrast, focal ischemia models emphasize acute injury responses and secondary neuroplastic remodeling following stroke, while microinfarction models simulate cumulative subclinical vascular insults contributing to cognitive vulnerability. Accordingly, mechanistic findings from these models should be interpreted as model-specific contributions to PSCI-relevant pathological cascades, rather than direct equivalents of clinical PSCI mechanisms. The convergence of findings across distinct models nevertheless supports the robustness of shared pathways—such as neuroinflammation, oxidative stress, and impaired network connectivity—that are consistently modulated by acupuncture.

This review did not include a formal risk-of-bias assessment, which may limit the ability to weigh the relative strength of individual studies. Given the heterogeneity of experimental models, clinical designs, and outcome measures, mechanistic conclusions should be interpreted with caution. Moreover, variability in acupoint selection, stimulation parameters, and intervention duration complicates cross-study comparisons and may contribute to inconsistent findings across the literature.

Despite accumulating mechanistic evidence, current research on acupuncture for PSCI still faces several limitations. First, heterogeneity in experimental design—such as acupoint selection, stimulation parameters, and intervention duration—complicates cross-study comparisons. Second, many studies rely on single-dimensional indicators without integrating molecular and imaging outcomes, limiting mechanistic depth. Third, translational gaps remain between animal and human studies, particularly regarding dose–response relationships and longitudinal neuroplastic effects.

Future research should focus on multi-omics integration (proteomics, metabolomics, transcriptomics) and multimodal neuroimaging (fMRI, DTI, PET) to map acupuncture’s effects across biological scales. The incorporation of network neuroscience and advanced data-analytic frameworks, including machine learning, may further elucidate system-level mechanisms underlying cognitive recovery. International multicenter collaborations using standardized protocols will be essential to generate reproducible evidence and strengthen the global acceptance of acupuncture in post-stroke rehabilitation.

## 5 Conclusion

Acupuncture offers a promising multi-target approach for the prevention and treatment of post-stroke cognitive impairment. Through coordinated modulation of neurotransmitter balance, neuroimmune homeostasis, antioxidative defense, neurotrophic signaling, and large-scale brain network connectivity, acupuncture may promote neuroplastic remodeling and cognitive restoration. The integrative neurobiological framework proposed in this review unites traditional concepts of systemic regulation with modern evidence of neural repair, providing a comprehensive model for understanding how acupuncture facilitates recovery after stroke.

Future studies integrating molecular biomarkers, functional imaging, and individualized treatment strategies will be crucial to validate this multi-pathway model and to advance precision acupuncture in cognitive rehabilitation.
