# Correction: Integrative neurobiological mechanisms of acupuncture in post-stroke cognitive impairment: from neurotransmission to brain network remodeling

**DOI:** 10.3389/fneur.2026.1819914

**Published:** 2026-03-13

**Authors:** Wei Li, Lebin Liu, Weiwei Liu, Huiping Liu

**Affiliations:** Department of Rehabilitation Medicine, Hubei Rongjun Hospital, Wuhan, China

**Keywords:** acupuncture, BDNF signaling, brain network remodeling, neuroinflammation, neuroplasticity, neurotransmission, post-stroke cognitive impairment, systems neurobiology

The figure captions were in the wrong order in the PDF/HTML version of this paper. Figure 1 had the caption of Figure 2; Figure 2 had the caption of Figure 1. The order has now been corrected.

The original version of this article has been updated.

