# Supplementary material for: Integrative neurobiological mechanisms of acupuncture in post-stroke cognitive impairment: from neurotransmission to brain network remodeling
Source: Front Neurol. 2026 Feb 25;17:1744242. doi: 10.3389/fneur.2026.1744242 (PMC12975595; doi:10.3389/fneur.2026.1744242)
Supplement: Supplementary file 1 [file Data_Sheet_1.pdf]

Supplementary Table S1. Database-Specific Search Strategies

Database	Search Fields	Search Strategy	Limits / Notes
PubMed	Title/Abstract; MeSH Terms	(("acupuncture"[MeSH Terms] OR acupuncture[Title/Abstract] OR electroacupuncture[Title/Abstract] OR manual acupuncture[Title/Abstract]) AND ("post-stroke cognitive impairment"[Title/Abstract] OR "vascular cognitive impairment"[Title/Abstract] OR poststroke dementia[Title/Abstract] OR PSCI[Title/Abstract]) AND (mechanism[Title/Abstract] OR neuroplasticity[Title/Abstract] OR neurotransmitter[Title/Abstract] OR BDNF[Title/Abstract] OR inflammation[Title/Abstract] OR oxidative stress[Title/Abstract] OR network[Title/Abstract]))	Language: English; Species: All
Web of Science	Topic	(acupuncture OR electroacupuncture OR manual acupuncture) AND ("post-stroke cognitive impairment" OR "vascular cognitive impairment" OR poststroke dementia OR PSCI) AND (mechanism OR neuroplasticity OR neurotransmitter OR BDNF OR inflammation OR oxidative stress OR network)	Indexes: SCI-EXPANDED; Language: English
Embase	Title/Abstract; Emtree Terms	(acupuncture OR electroacupuncture OR manual acupuncture) AND ("post-stroke cognitive impairment" OR "vascular cognitive impairment" OR poststroke dementia OR PSCI) AND (mechanism OR	Language: English; Article type: Article

neuroplasticity OR neurotransmitter OR BDNF OR inflammation OR oxidative stress OR network)
